# LncRNA UCA1 Participates in De Novo Synthesis of Guanine Nucleotides in Bladder Cancer by Recruiting TWIST1 to Increase IMPDH1/2

**DOI:** 10.7150/ijbs.82875

**Published:** 2023-05-08

**Authors:** Shan-Shan Liu, Jia-Shu Li, Mei Xue, Wen-Jing Wu, Xu Li, Wei Chen

**Affiliations:** 1 Clinical Laboratory, The First Affiliated Hospital of Xi'an Jiaotong University, Xi'an, Shaanxi 710061, PR China.; 2 Center for Translational Medicine, The First Affiliated Hospital of Xi'an Jiaotong University, Xi'an, Shaanxi 710061, PR China; Key Laboratory for Tumor Precision Medicine of Shaanxi Province, The First Affiliated Hospital of Xi'an Jiaotong University, Xi'an, Shaanxi 710061, PR China.

**Keywords:** bladder cancer, UCA1, IMPDH, guanine nucleotide, metabolism

## Abstract

Metabolic dysregulation has been identified as one of the hallmarks of cancer biology. Based on metabolic heterogeneity between bladder cancer tissues and adjacent tissues, we discovered several potential driving factors for the bladder cancer occurrence and development. Metabolic genomics showed purine metabolism pathway was mainly accumulated in bladder cancer. Long noncoding RNA urothelial carcinoma-associated 1 (LncRNA UCA1) is a potential tumor biomarker for bladder cancer diagnosis and prognosis, and it increases bladder cancer cell proliferation, migration, and invasion via the glycolysis pathway. However, whether UCA1 plays a role in purine metabolism in bladder cancer is unknown. Our findings showed that UCA1 could increase the transcription activity of guanine nucleotide de novo synthesis rate limiting enzyme inosine monophosphate dehydrogenase 1 (IMPDH1) and inosine monophosphate dehydrogenase 2 (IMPDH2), triggering in guanine nucleotide metabolic reprogramming. This process was achieved by UCA1 recruiting the transcription factor TWIST1 which binds to the IMPDH1and IMPDH2 promoter region. Increased guanine nucleotide synthesis pathway products stimulate RNA polymerase-dependent production of pre-ribosomal RNA and GTPase activity in bladder cancer cells, hence increasing bladder cancer cell proliferation, migration, and invasion. We have demonstrated that UCA1 regulates IMPDH1/2-mediated guanine nucleotide production via TWIST1, providing additional evidence of metabolic reprogramming.

## Introduction

The world's tenth malignant tumor, bladder cancer, is a major threat to health and human life 1. The lack of molecular knowledge regarding the pathogenicity of the disease hampers the development of clinical bladder cancer treatment 2. Cancer often features metabolic abnormalities as one of its primary hallmarks 3, and thus it is possible to comprehend how the disease develops by studying the control mechanisms of aberrant metabolism in bladder cancer.

Unbalanced purine nucleotide metabolism is a major factor in the emergence and progression of cancers and encourages their growth 4. Purine nucleotides include adenine nucleotides and guanine nucleotides, and enzyme expression in the guanine nucleotide synthesis pathway is strongly activated in tumor cells 5. As enzymes express strongly, metabolites rise. This provides more energy for physiological processes such as energy transport, signal transduction, and protein synthesis, which leads to tumor cells displaying malignant behavior. The rate-limiting enzymes for de novo guanine synthesis, inosine monophosphate dehydrogenase 1 (IMPDH1) and inosine monophosphate dehydrogenase 2 (IMPDH2), catalyze IMP to create xanthate 5'-monophosphate (XMP) 6. Prostate cancer 7, breast cancer 8 and lung cancer 9 progress due to abnormal guanine nucleotide synthesis caused by abnormal expression of IMPDH1 and IMPDH2. In bladder cancer, however, the mechanisms underlying metabolic imbalances remain largely unknown.

Research on bladder cancer mainly focuses on abnormal glucose metabolism. Bladder cancer cells circumvent apoptosis by rearranging glutamine metabolism and abnormal metabolic glycolysis to promote proliferation and migration 10-12, this process is primarily regulated by long non coding RNAs (lncRNAs) 13, 14. The lncRNA urothelial carcinoma-associated 1 (UCA1) was elevated in bladder cancer tissues, according to our prior work. In nude mice, overexpression of UCA1 promotes the proliferation, migration, invasion, anti-apoptosis, tumorigenicity, and treatment resistance of bladder cancer cells 15-18. In bladder cancer cells, UCA1 regulates the hexokinase glycolytic enzyme 2 (HK2), which significantly enhances aerobic glycolysis 19, 20, and it participates in the metabolic remodeling of cancer cells 21. UCA1 upregulates glutamate pyruvate transaminase 2 (GPT2) expression through interaction with heterogeneous nuclear ribonucleoprotein I/L, and promotes glutamine driven bladder cancer reperfusion 22. There are reports that the concentration of purine nucleosides in the urine of bladder cancer patients is higher than that of normal people's urine 23. However, there is still little information on how UCA1 affects guanine nucleotide metabolism in bladder cancer.

One of the processes regulating guanine nucleotide biosynthesis may involve UCA1, which recruits transcription factors to enhance the expression of guanine nucleotide biosynthesis enzymes. Different transcription factors can produce nucleotides at the level of gene expression 24. For instance, the transcription factor c-Myc controls nucleotide production by increasing the expression of many nucleotides 25, 26. Thymidylate synthase (TS), IMPDH1, and IMPDH2, and other nucleotide biosynthesis enzymes can be expressed more frequently when lncRNA upregulated c-Myc 27, 28. The transcription factor TWIST1 enhances the malignant potential of bladder cancer by increasing tumor treatment resistance, proliferation, and metastasis in human bladder tumor tissues 29. There are few reports on the role of TWIST1 in the metabolism of bladder cancer. Exploring the mechanism of TWIST1 participating in guanine nucleotide synthesis in bladder cancer expands the role of TWIST1 in bladder cancer.

In conclusion, the purpose of this study is to examine how UCA1 regulates guanine nucleotides to facilitate bladder cancer progression in bladder cancer cells. By providing new evidence for understanding the purine metabolism in bladder cancer cells and lncRNAs regulatory role in tumor cell metabolic remodeling, we hope to create medications that specifically target bladder cancer by using the findings of this study.

## Materials and Methods

### Cell lines

Human bladder cancer cell lines were cultured in RPMI-1640 (Gibco # 31800-022) containing 10% fetal bovine serum. The cell culture temperature was 37℃ and the gas condition was 5% CO2. Using X-tremeGENE according to the manufacturer's specifications, HP DNA Transaction Agent (Roche#06366236001) transfected the plasmid into cells. Stable UCA1 knock-down 5637 cells (HTB-9) were transfected with pRNAT-U6.1/Neo-Nc and pRNAT-U6.1/Neo-shUCA1.1 plasmid, respectively 18. The stable cell lines with UCA1 overexpression and their control in UMUC2 cells (CRL-1748) were named pcDNA-U and pcDNA-M 30. Continuous incubation for 48 hours can be applied to detect RNA and 72 hours to detect protein levels. The knockdown or overexpression cell lines of IMPDH1/2 were constructed by lentivirus infection. When the cell fusion degree reached 20% in UMUC2 or 5637 cells in a 6-well plate (for around 12 hours), we replaced the culture medium with 1ml of fresh culture medium containing 1× HiTransG P virus infection reagent (GENE#REVG005), and added the virus suspension. After 48 hours of culture, cells were screened using a medium containing 2μg/ml puromycin, and the medium was renewed every 2 days. After about 1-week, stable infected cell lines were obtained. The sequences involved in plasmids and viruses are shown in Supplementary Table 1.

### Mouse Studies

3-4-week-old male BALB/c-mice were purchased from Gempharmatech Co., Ltd. and raised in a specific pathogen free animal room. The Animal Ethics Committee of Xi'an Jiaotong University approved the xenograft procedure. 3×10^6^ cells were inoculated into the axillae of BALB/c- mice, and nude mice's tumor growth and body weight were monitored every 2 days. Tumor volume was approximately calculated based on the long and short diameters: volume = a^2^b/2 (a: short diameter, b: long diameter). About twenty days later, BALB/c-mice were dislocated and dissected after deep coma and finally weighed the weight of tumor tissue in nude mice.

### Metabolomics

Cells were incubated in RPMI 1640 medium labeled with ^15^N glutamine (MilliporeSigma # 490024) or without isotope glutamine. Cells digested with pancreatin solution and resuspended in cold PBS solution were counted by cell counter, resuspended with 80% cold acetonitrile, ultrasonically separated, and extracted. 2010 AB SCIEX was used to conduct targeted metabonomic analysis of the supernatant with a triple quadrupole liquid chromatography-mass spectrometer (API3200 LC-20A). The injection volume was 10μl, gradient program: 0-0.1 min 5% B; 0.1-1 min 80% B; 1-1.5 min 5% B; 1.5 min-3 min 5% B maintenance. Chromatogram review and peak area were integrated using Applied Biosystems SCIEX. The total area was calculated by manual integration, and the standard concentration of the test substance was used for the quantitative analysis.

### qRT-PCR

Total RNA was extracted from cells, BALB/c-tissue or patient tissue and isolated using TRIzol (Invitrogen #15596018). cDNA was generated with the PrimeScript RT kit (Takara #RR047A). TB Green Premix Ex Taq™ II (Takara #RR820A) measured relative cDNA abundance, and β-actin or GAPDH as an internal reference gene for target genes. The relative expression of target genes was calculated through the 2-^ΔΔCT^ (ΔCT=CT_target_-CT_control_) method and normalized to the relative expression tested in control cells (set to 1.0). For more than two gene analysis between two groups, we used 2^-ΔCT^ (ΔCT=ΔCT_tumour_-ΔCT*_β_*_-actin_) method to calculate the gene expression level (defined as multiple changes). The primer sequences are listed in Supplementary Table 1.

### Western blotting

RIPA (Beyotime#P0013B) supplemented with phosphatase inhibitor II (ZHHC#PL026) and PMSF (ZHHC#PL012) were used to lyse bladder cancer cells. Protein concentration was determined by BCA (Yamei#ZJ101L) and then separated into 10% SDS-PAGE gels with antibodies for bound protein and ECL blotting system (GLPbio#GK10008) to detect the bands. Primary antibodies were used in the experiments included: β-actin (1:2000; Proteintech#81115-1-RR); GAPDH (1:2000; Proteintech#10494-1-AP); IMPDH1 (1:3000; Proteintech#22092-1-AP); IMPDH2 (1:4000; Abcam#ab131158); TWIST1 (1:70; Santa cruz#sc-81417/ 1:1000; Proteintech#25465-1-AP); NFIL3 (1:150; Santa cruz#sc-74415); IKZF1 (1:150; Santa cruz#sc-398265); TEAD2 (1:150; Santa cruz#sc-81397); GLI2 (1:150; Santa cruz#sc-271786); PRRX1 (1:150; Santa cruz#sc-293386); FLI1 (1:150; Santa cruz#sc-365294); MMP9 (1:150; Santa cruz#sc-13520); PCNA (1:200; Santa cruz#sc-56); MMP2 (1:150; Santa cruz#sc-13595); EMMPRIN (1:150; Santa cruz#sc-21746).

### Chromatin immunoprecipitation (ChIP) assay and RNA immunoprecipitation (RIP)

Using the Magna Chip A/G Kit (Merck/17-0085), Chip was performed as instructed and the 1×10^7^ cells were subjected to cross-linked ultrasound to form fragments of 200-500 base DNA, and the ultrasound-induced cell lysates were incubated overnight using A/G magnetic beads bound to anti-human TWIST1 antibody, and the rabbit lgG was used as a negative control. Finally, the fragment was purified by the QIAquick PCR Purification kit and the QIAquick PCR & Gel Cleanup kit (QIAGEN#28104). qPCR and RNA gel electrophoresis were used to determine the abundance of binding sites. The specific primers required for ChIP-qPCR experiment are shown in Supplementary Table 1.

We used the Magna RIP kit (Merck/17-700) for RIP as per manufacturer's guidelines. The TWIST1 antibody of mouse anti human added was set as the experimental group, and the mouse IgG as the negative control. The cell lysate and RIP buffer containing magnetic beads were stirred overnight at 4°C. The qRT-PCR and RNA gel electrophoresis were used to detect the coprecipitated RNA binding protein RNA complex, and the total RNA (input) and IgG control were measured simultaneously.

### RNA pull down

Using full-length linear DNA templates of UCA1 amplified by primers containing T7 promoter and the RNAmax-T7 kit (Ribo/C11001-1), the sense, and antisense strands of UCA1 were transcribed in vitro, and the DNA templates were removed. The sense and antisense strands of UCA1 were labeled with biotin using the Pierce RNA 3'End Desthiobiotinylation Kit (Thermo scientific/20163). Cells were collected and lysed. By using streptavidin magnetic beads from the Pierce magnetic RNA pull-down kit (Thermo scientific/20164), the biotin-labeled UCA1 probes were captured and incubated with purified proteins. Dropdown ribosome binding proteins were analyzed by western blotting.

### Promoter luciferase assay

The genomic region containing the wild-type or mutant allele of the IMPDH1 or IMPDH2 promoter was cloned into the pGL4.11 construct. 1.5 μg of reporter construct was co-transfected with 50 ng of pRL-SV40-Renilla luciferase into 5637 cells using HP DNA transfection reagent. Cells were harvested at 24 hours, and luciferase activity was measured according to the procedure of the Dual Luciferase Reporter Assay System (Promega #E1910).

### Cell proliferation, migration, and invasion

To assess cell proliferation rates, cells were inoculated in 96-well plates with 1200 cells per well, and the Cell Counting Kit-8 (CCK8) measured absorbance at 450 nm every 24 hours for three days. By measuring the proportion of red fluorescent cells labeled, the BeyoClick EdU-594 Cell Proliferation Assay Kit assay indicated the proliferation status of cells. For the invasion assay, transfer well inserts were coated with matrix gel (ABW#0827065) and 1.2×10^5^ cells in serum-free medium were plated on top of the coated filter. Complete medium was placed in the lower chamber. A cotton swab was used to remove cells that had not migrated or invaded the perforated inserts after 24 hours, and it was fixed and dyed with methanol and crystal violet. 5×10^4^ cells were placed into the upper lumen of the insert (8 mm; BD Bioscience), and determined the migration ability of cells by calculating the number of cells migrating to the lower ventricular surface.

### Immunohistochemistry

48 patients with bladder cancer or adjacent tissues were collected from the First Affiliated Hospital of Xi'an Jiaotong University, Xi'an, China (Supplementary Table 2). All samples were collected and operated following the ethical requirements of the Institutional Review Committee of the First Affiliated Hospital of Xi'an Jiaotong University. IHC detected expression of TWIST1, IMPDH1, and IMPDH2 proteins. Primary antibodies included rabbit monoclonal antibody IMPDH1 (1:200; 22092-1-AP; Proteintech); IMPDH2 (1:200; ab131158; Abcam); TWIST1 (1:200; 25465-1-AP; Proteintech); murine monoclonal antibody ki67 (1:400; GB121141; Servicebio technology). IHC was performed as follows: 4 µm thick tissue sections were cut, dewaxed in xylene, hydrated with graded ethanol and the sections were then repaired with thermal antigen. Secondary antibodies were purchased from ZSGB-BIO (SP-9001 and SP-9002, Beijing, China). All tumor cells were quantified as percentages of positive cells for protein expression (range 0-100%).

## Results

### UCA1 regulates the proliferation, migration, and invasion of bladder cancer cells through IMPDH1/2

**Figure 1:**
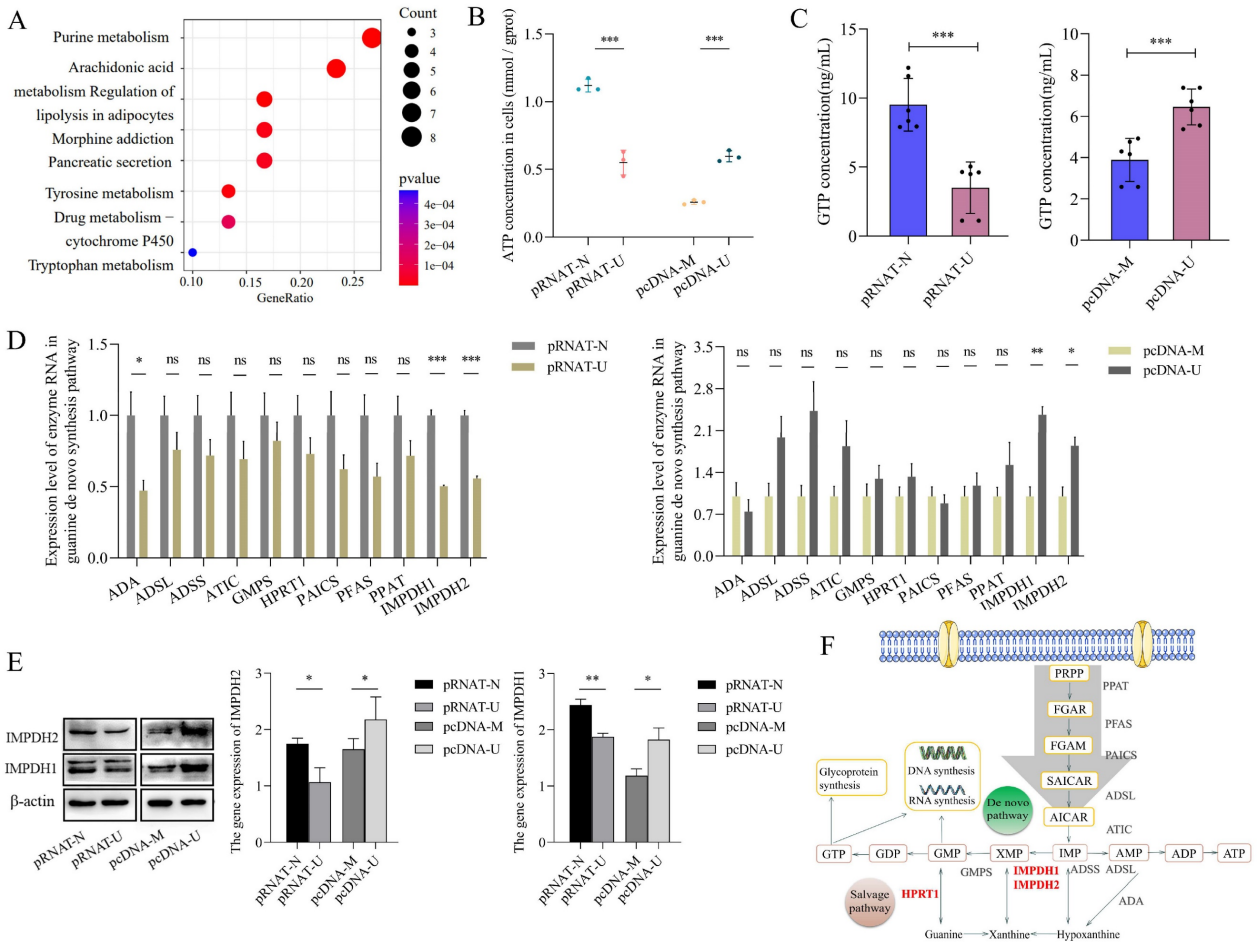
UCA1 regulates IMPDH1/2 involvement in guanine nucleotide de novo synthesis. A, Analysis of metabolic pathways of differential expression between bladder cancer and adjacent tissues by TCGA database. B, ATP levels in cellular. UCA1 knockdown group (control: pRNAT-N; experimental: pRNAT-U) and UCA1 overexpression group (control: pcDNA-M; experimental: pcDNA-U). C, GTP levels in cellular. D, Purine nucleotide synthesis-related enzyme expression were detected by qRT-PCR. E, Western blotting results of IMPDH1 and IMPDH2 proteins expression were found in UCA1 knockdown cells or UCA1 overexpression cells. F, Purine synthesis pathway. Red font indicates rate-limiting enzyme. Data are expressed as mean ± SEM for qRT-PCR and mean ± SD for other analyses. ns, no significance. All experiments were repeated more than three times. ns, no significance; * p<0.05; **p<0.01; ***p<0.001.

Bladder cancer is characterized by metabolic abnormalities. In this study, GSEA enrichment analysis found that nucleic acid, lipid, carbohydrate, and other metabolic pathways were enriched in bladder cancer species. (Supplementary Figure 1). Further analysis found that purine metabolism was the most significant metabolic pathway between cancer and adjacent tissues in the TCGA database of bladder cancer (Figure 1A).

Our previous research confirmed that UCA1 is involved in bladder cancer cells' glucose and glutamine metabolism 15-18. To study the role of UCA1 in purine synthesis, we constructed a stable transfected cell line that knocks down UCA1 in 5637 cells and a stable transfected cell line that overexpresses UCA1 in UMUC2 cells (Supplementary Figure 3A). Then we detected ATP and GTP concentration (purine metabolites) in 5637 and UMUC2 cells, we found that the levels of ATP and GTP in cells were positively correlated with the expression of UCA1 (Figure 1B, C). Therefore, UCA1 may promote the biosynthesis of purine.

In order to determine whether UCA1 plays a role in purine synthesis in bladder cancer, we analyzed the enzymes involved in the purine synthesis pathway (including guanine nucleotide synthesis and adenine nucleotide synthesis). It was found that the expression level of most enzymes was positively correlated with the level of UCA1, but only IMPDH1 and IMPDH2 were statistically significant (Figure 1D). At the protein level, we verified that UCA1 regulates the expression of IMPDH1 and IMPDH2 (Figure 1E), and the enzymes in the pathway role was shown in Figure 1F. UCA1 regulates the rate-limiting enzyme IMPDH1/2 of guanine nucleotide de novo synthesis.

**Figure 2:**
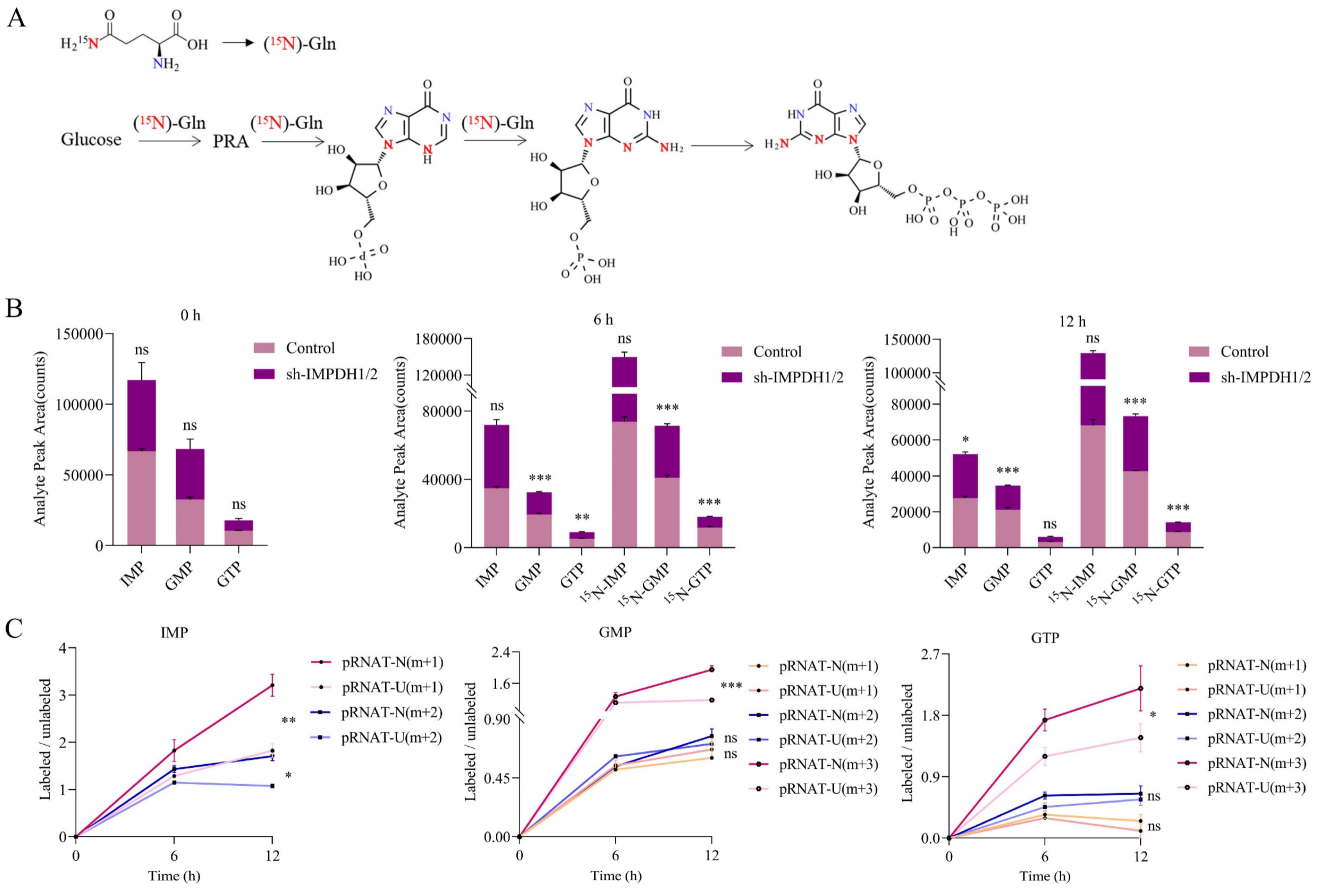
Detection of the metabolic rate of guanine nucleotides by isotope tracer. A, Possible locations of molecules labeled after isotopic tracing. B, Comparison the metabolites of IMP, GMP and GTP between labeled group and unlabeled group. C, The effect of knocking down UCA1 on the levels of labeled metabolites. All experiments were repeated more than three times. ns, no significance; * p<0.05; **p<0.01; ***p<0.001.

In order to confirm that UCA1 regulates guanine nucleotide biosynthesis through IMPDH1/2, we used an isotope tracer. We added ^15^N-labelled (amide-^15^N) glutamine to the glutamine medium. In addition to the nitrogen contained in phosphoramide, it provides additional amide nitrogen for IMP. After 6 hours or 12 hours incubation, (amide ^15^N) glutamine-labeled IMP, GMP, and GTP increased. The location of the possible markers is shown in the figure (Figure. 2A). A reduction in the ratio of labeled GMP and labeled GTP levels was observed in IMPDH1/2 knockdown cells at 6 hours and 12 hours, indicating a reduction in guanine nucleotide synthesis (Figure 2B). Metabolite levels of IMP m+1, IMP m+2, GMP m+3 and GTP m+3 was lower in UCA1 knockdown cells than in controls after 12 hours of isotope tracer labeling (Figure 2C). Isotopic tracer showed that UCA1 affected the metabolites of guanine nucleotide de novo synthesis pathway.

### IMPDH1/2 promotes proliferation, migration, and invasion of bladder cancer cells by up-regulating GTPase activity and the RNA polymerase-dependent expression of pre-ribosomal RNA

**Figure 3:**
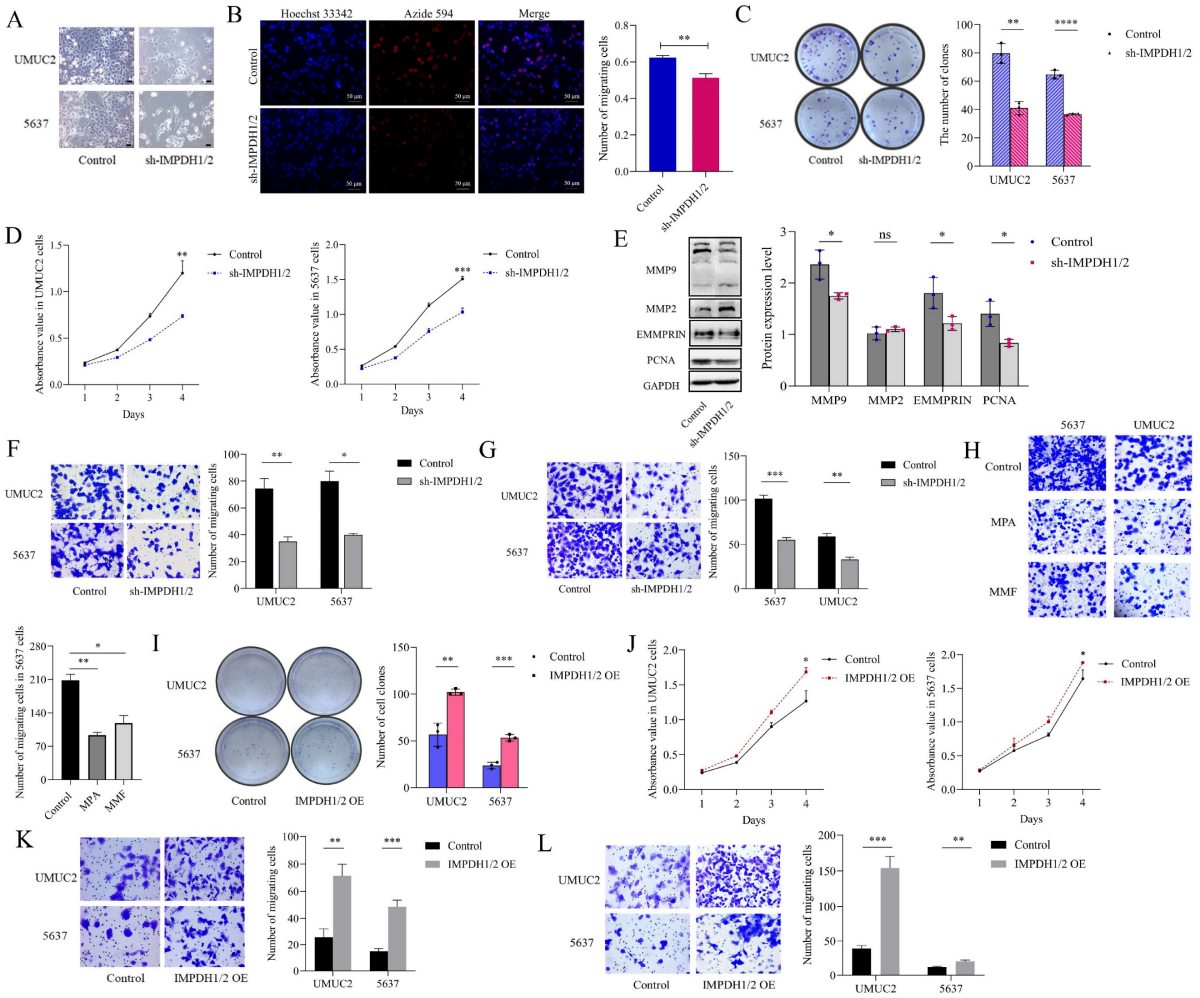
IMPDH1/2 promotes proliferation, migration, and invasion of bladder cancer cells. A, Morphological changes in IMPDH1/2 knockdown cells. Scale bar = 20μm. B, The proliferation of IMPDH1/2 knockdown cells was detected by EdU assay. C, A colony formation experiment was used to identify the colony formation ability of IMPDH1/2 knockdown cells. D, CCK8 assay was used to detect IMPDH1/2 knock-down cell proliferation. E, Western blotting was used to detect the proliferation, migration, and invasion-associated proteins normalized to GAPDH. F, G, The Transwell assay showed that knockdown of IMPDH1/2 inhibited the migratory and invasion abilities of 5637 and UMUC2 cells. H, Transwell assays were used to detect the migration abilities of cells treated with MPA and MMF. I, A colony formation experiment was used to identify the colony formation ability of IMPDH1/2 overexpression cells. J, CCK8 assay tested the effect of IMPDH1/2 overexpressed on cellular proliferation in 5637 and UMUC2 cells. K, L, Transwell assay showed migration and invasive capacity of IMPDH1/2 overexpressed cells. The data are expressed as mean ± SD. All experiments were repeated more than three times. ns, no significance; * p<0.05; **p<0.01; ***p<0.001; ****p<0.0001.

To examine the effect of IMPDH1 and IMPDH2 on bladder cancer cell's malignant behavior, we constructed stable cell lines knockdown IMPDH1/2 in 5637 cells and UMUC2 cells (Supplementary Figure 3B and C). We found that the cell morphology was flat, the cell edge was rough, and the number of cell fragments in the culture medium was increased (Figure 3A). 5-ethynyl-20-deoxyuridine (EdU), CCK-8, and plate cloning experiments showed that cell proliferation decreased (Figure 3B, C, D). A decrease in MMP9, EMMPRIN, and PCNA protein inhibited cell proliferation, invasion, and migration (Figure 3E). Transwell assay confirmed that the cell migration and invasion ability were reduced (Figure 3F, G). When treated with IMPDH inhibitors MPA and MMF, bladder cancer cells showed reduced migration ability (Figure 3H). On the contrary, we constructed stable cell lines overexpression IMPDH1/2 in 5637 cells and UMUC2 cells (Supplementary Figure 3D and E). Overexpression of IMPDH and plate cloning showed increased cell proliferation (Figure 3I). CCK8 assay showed an increase in cell proliferation (Figure 3J), and Transwell analysis showed an increase in cell migration and invasion (Figure 3K, L). These results indicated that IMPDH1 and IMPDH2 promote bladder cancer cell proliferation, migration, and invasion.

**Figure 4:**
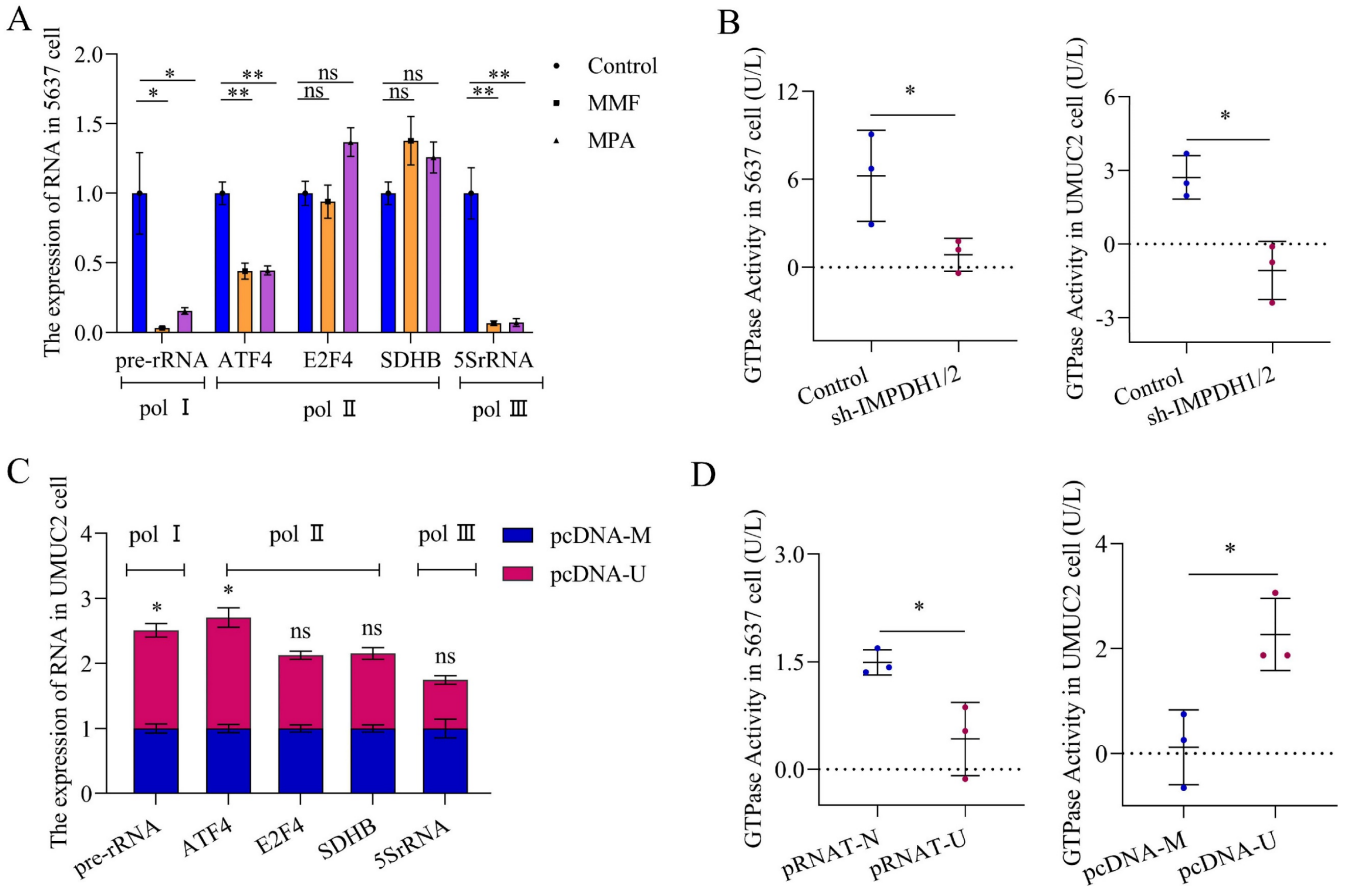
The mechanism of IMPDH1/2 regulates bladder cancer. A, pre-rRNA level following inhibitor treatment. B, GTPase activity assay in cells of IMPDH knockdown. C, pre-rRNA expression in UCA1 overexpressed cells. D, GTPase activity in UCA1 knockdown cells. Data are expressed as mean ± SEM for qRT-PCR and mean ± SD for other analyses. All experiments were repeated more than three times. ns, no significance; * p<0.05; **p<0.01.

We studied the mechanism of IMPDH1/2 promoting the proliferation, migration, and invasion of bladder cancer cells. Changes in IMPDH1 and IMPDH2 expression cause abnormal guanine biosynthesis, affecting tumor cells physiologically. We used MPA and MMF (inhibitors of IMPDH1 and IMPDH2) to measure the inhibitor concentration with a 50% proliferation inhibition rate and treated the cells (Supplementary Figure 2A), and pre-rRNA level was decreased (Figure 4A). When knocking down IMPDH in 5637 and UMUC2 cells, GTPase activity also decreased (Figure 4B). Further examination of the effect of UCA1 on pre-rRNA and GTPase activity, it was found that UCA1 promoted pre-rRNA expression and GTPase activity (Figure 4C, D). In other words, UCA1 affects the expression of pre-rRNA and GTPase activity by regulating IMPDH1 and IMPDH2, thus promoting the malignant behavior of bladder cancer.

### UCA1 regulates IMPDH1/2 via recruiting TWIST1

**Figure 5:**
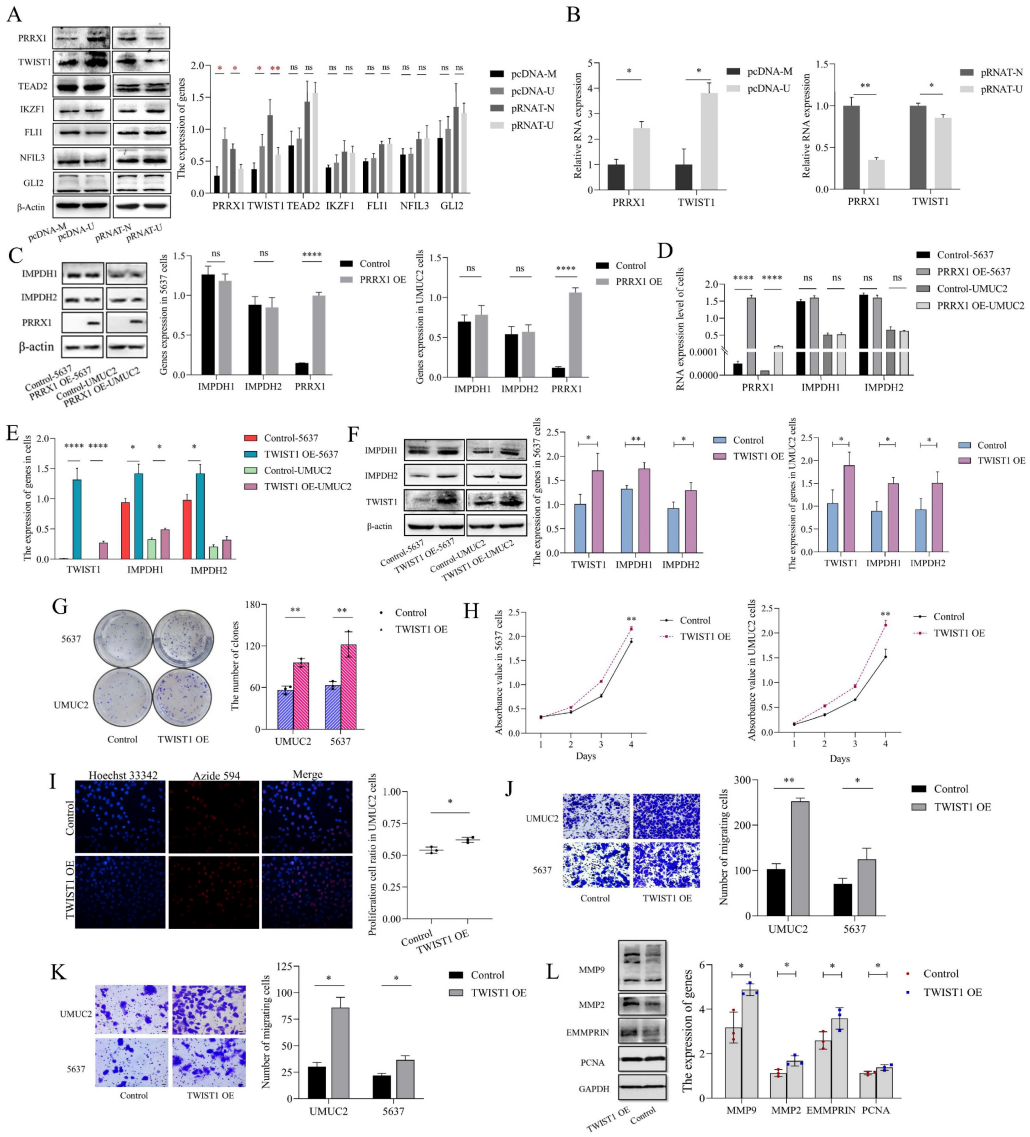
UCA1 regulates the expression of IMPDH1/2 through TWIST1. A, Western blotting was used to detect the transcription factor proteins normalized to β-actin. B, Transcription factors expression levels were detected by qRT-PCR. C, The expression levels of IMPDH1 and IMPDH2 were examined using western blotting in PRRX1 overexpressed cells. D, qRT-PCR detected IMPDH1 and IMPDH2 expression in PRRX1 overexpressed cells. E, qRT-PCR detected IMPDH1 and IMPDH2 expression in TWIST1 overexpressed cells. F, The expression levels of IMPDH1 and IMPDH2 were examined using western blotting in TWIST1 overexpressed cells. G, A colony formation experiment was used to identify the colony forming ability of TWIST1 overexpression cells. H, CCK8 assay determined the proliferation of TWIST1 overexpressed cells. I, The proliferation of TWIST1 overexpressed cells detected by EdU assay. J, K, Transwell assay showed that invasion and migration capacity of TWIST1 overexpressed cells. L. Western blotting detected proliferation, migration, and invasion-associated proteins (MMP9, MMP2, EMMPRIN, and PCNA) in TWIST1 overexpressed cells. Data are expressed as mean ± SEM for qRT-PCR and mean ± SD for other analyses. All experiments were repeated more than three times. ns, no significance; * p<0.05; **p<0.01; ***p<0.001; **** p<0.0001.

To investigate the mechanisms by which UCA1 regulates IMPDH1/2, we predicted the potential transcription factors (Supplementary Table 3) and screened seven genes (PRRX1, TWIST1, TEAD2, IKZF1, FLI1, NFIL3, and GLI2). According to western blotting experiments, knockdown or overexpression of UCA1 reduced or increased PRRX1 and TWIST1 expression, and the expression of TEAD2, IKZF1, FLI1, NFIL3, and GLI2 genes were not significantly different (Figure 5A, B). However, overexpression of PRRX1 did not affect IMPDH1 and IMPDH2 expression (Figure 5C, D), suggesting that PRRX1 does not regulate the expression of IMPDH1 and IMPDH2. Overexpression of TWIST1 increased the expression of the downstream genes IMPDH1 and IMPDH2 (Figure 5E, F). Overexpression of TWIST1 with the unchanged expression of UCA1 (Supplementary Figure 2B). IMPDH1/2 was overexpressed, and UCA1 and TWIST1 expression was not statistically significant (Supplementary Figure 2C, D). The knock-down of IMPDH1/2 did not cause the difference in the expression of UCA1 and TWIST1 (Supplementary Figure 2E). Cancer cells also proliferate, migrate, and invade when TWIST1 was activated. The overexpression of TWIST1 causes the malignant increase of bladder cancer cells. Plate cloning analysis, EdU analysis, and CCK8 assays showed enhanced cell proliferation (Figure 5G, H, I), migration and invasion analysis showed enhanced cell migration and invasion (Figure 5J, K), and western blotting analysis showed increased expression of MMP9, MMP2, PCNA, and EMMPRIN proteins (Figure 5L). Based on the above results, UCA1 regulates IMPDH1/2 via the UCA1/TWIST1/IMPDH axis, and there is no feedback loop formed between molecules.

**Figure 6:**
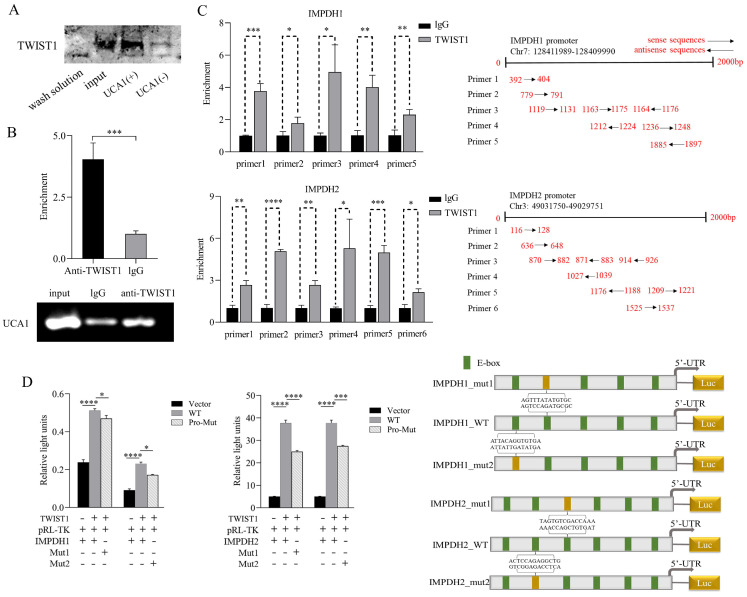
Validation of binding sites for UCA1, TWIST1, and IMPDH1/2. A, RNA pull-down confirmed the enrichment of TWIST1 protein in each group through western blotting. B, Immunoprecipitation of UCA1 was performed in 5637 cells, followed by PCR and RT-qPCR to test the number of bindings of UCA1 to TWIST1. C, ChIP assays were performed in 5637 cells to appraisal the binding sites of TWIST1 to the promoters of IMPDH1 and IMPDH2 (calculated by IP/input). The schematic on the right indicates the specific sequence of each primer in the promoter region. D, Dual-luciferase reporter assay identified the interaction of TWIST1 with IMPDH1 and IMPDH2. The TWIST1 fragment with binding sites to the IMPDH1/2 promoter region was cloned into a firefly luciferase pGL3-control vector. Luciferase activity was standardized by co-transfected the Ranila luciferase plasmid. Green squares on the right-hand schematic represent binding sites, and yellow squares represent mutation sites. Data are expressed as mean ± SEM for qRT-PCR and mean ± SD for other analyses. ns, no significance; * p<0.05; **p<0.01; ***p<0.001; **** p<0.0001.

To prove the binding sites of the pathway molecules, RNA pull-down experiment was performed with the UCA1 sense chain and antisense chain containing the T7 promoter. Compared with the antisense chain, the UCA1 sense sequence pulled down more TWIST1 protein (Figure 6A), indicating a binding site between UCA1 and TWIST1. In the RIP experiment, RNA was extracted using an anti-TWIST1 antibody and UCA1 expression was detected and analyzed. Compared with the control IgG immune precipitate, UCA1 was specifically enriched in the RNA pulled down by TWIST1 protein (Figure 6B). In conclusion, we clarified that there was a binding site between UCA1 and TWIST1. The jaspar database was used for ChIP analysis to predict the possible binding sites of TWIST1-IMPDH1 and IMPDH2 involved in transcriptional activation. Pull down DNA fragments using TWIST1 antibody showed that IMPDH1 and IMPDH2 were enriched at multiple sites (Figure 6C), which proves that TWIST1 plays an important role in IMPDH1 and IMPDH2. In a dual-luciferase reporter gene experiment, TWIST1 strongly promoted the transcription of downstream IMPDH1 and IMPDH2. We selected two sites of IMPDH1 and IMPDH2 that bind to TWIST1 to construct mutant plasmids. One mutation site partially reduced the promotion of TWIST1 on IMPDH1 and IMPDH2 compared with the wild type (Figure 6D), confirming our CHIP results, that is, TWIST1 regulates IMPDH1 and IMPDH2 promoters at multiple sites.

### Rescue experiment of molecule and nucleotide on the cell's biological ability

**Figure 7:**
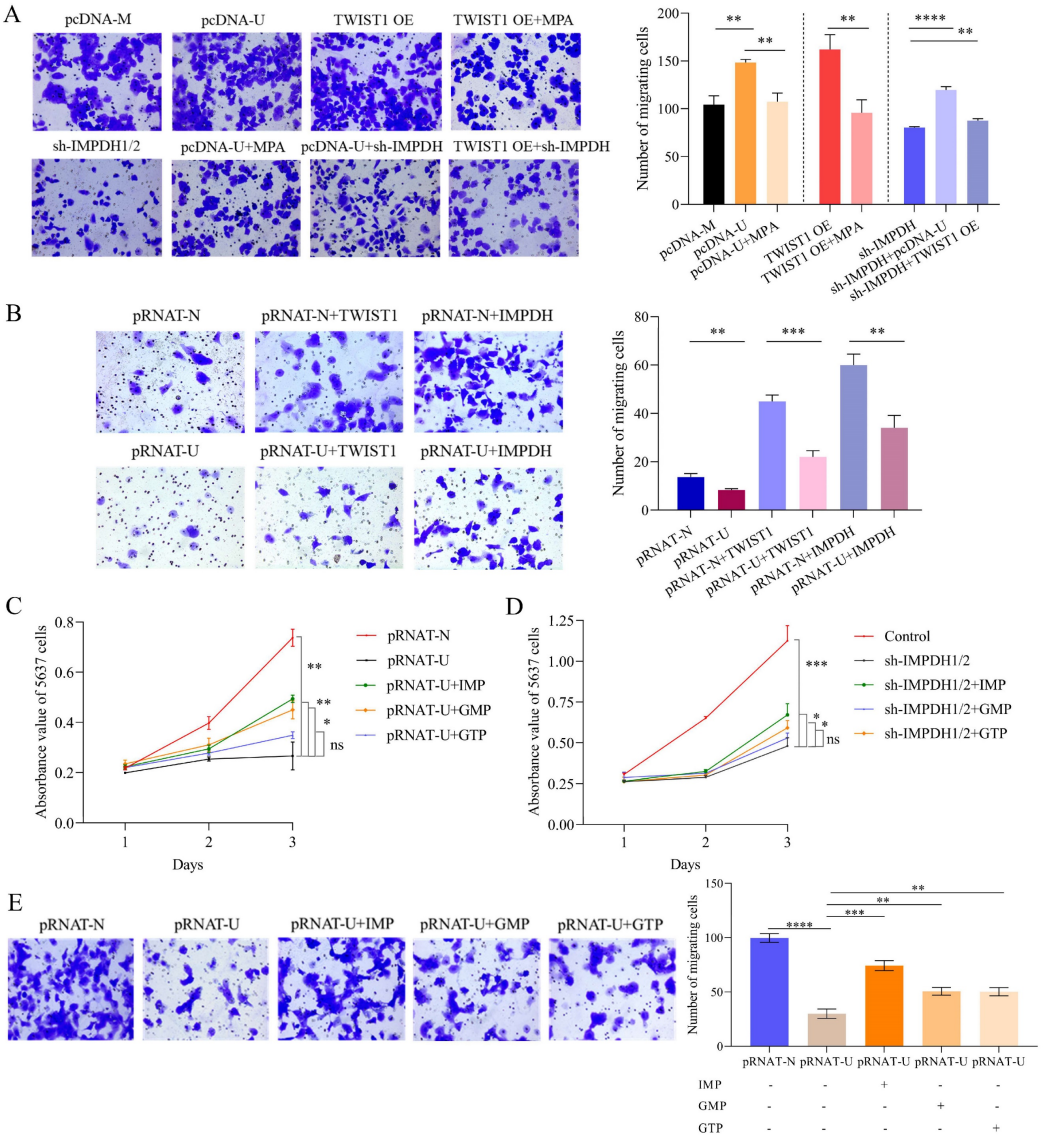
Phenotype recovery of bladder cancer cells. A, Transwell migration experiment detected the migration abilities of UMUC2 cells. B, Transwell migration experiment detected the migration abilities of 5637 cells. C, CCK8 indicated that supplemented with IMP, GMP, or GTP rescued the proliferation ability of UCA1 knockdown cells. D, CCK8 indicated that supplemented with IMP, GMP, or GTP rescue the proliferation ability of IMPDH1/2 knockdown cells. E, Transwell assay showed that supplementation with IMP, GMP, and GTP facilitate the migratory abilities of UCA1 knockdown cells. The data are expressed as mean ± SD. All experiments were repeated more than three times. ns, no significance; * p<0.05; **p<0.01; ***p<0.001; **** p<0.0001.

The bladder cancer cell phenotype can be recovered by controlling molecules or supplementing corresponding nucleotide analogues. Bladder cancer cells overexpressing UCA1 or TWIST1 had reduced migratory capacity after MPA treatment or knockdown of IMPDH1/2 (Figure 7A). Overexpression of TWIST1 or IMPDH1/2 in 5637 cells knockdown UCA1 enhanced migration of bladder cancer cells (Figure 7B). In the group of knocked down UCA1 or IMPDH, 50μM of IMP, GMP, and GTP were supplemented 31, respectively. The supplemented IMP and GMP cells showed an increase in proliferation rate, but did not fully recover to the control level (Figure 7C, D). The cell migration analysis also confirmed an increase in cell migration following supplementation with IMP, GMP, and GTP, the least increase was seen in the GTP-supplemented group, and IMP supplementation resulted in the best cellular migration reversion (Figure 7E). The nucleotide supplementation experiments fully demonstrated that UCA1 affected the metabolites IMP, GMP, and GTP levels simultaneously and that GTP supplementation alone did not reverse the tumor cell phenotype. The rescue experiment verified the regulatory of UCA1 on guanine nucleotide in bladder cancer cells.

### Verification experiment of UCA1/TWIST1/IMPDH axis in vivo

**Figure 8:**
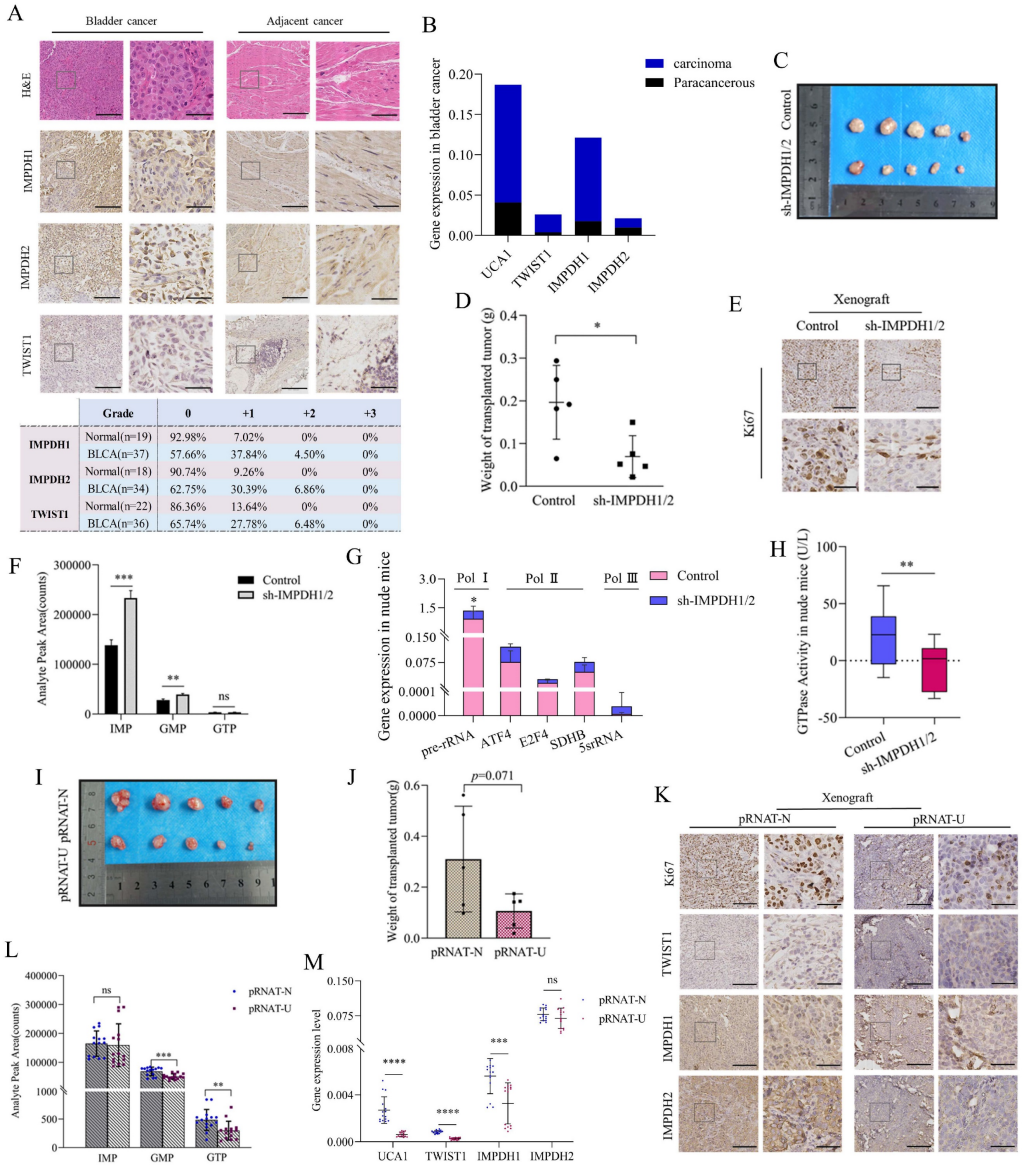
Validation UCA1/TWIST1/IMPDH axis in vivo. A, Quantitative analysis of immunohistochemical of TWIST1, IMPDH1, and IMPDH2 proteins in clinical specimens. B, UCA1, TWIST1, IMPDH1, and IMPDH2 expression levels in clinical tissue were detected by qRT-PCR. C, D, Tumor morphology and weight of nude mice tumor tissue with knockdown IMPDH1/2. E, Immunohistochemical detected ki67 expression in nude mice tumor tissue of IMPDH1/2 knockdown. F, The metabolite levels in nude mice tumor tissue of IMPDH1/2 knockdown. G, The expression level of RNA polymerase dependent RNA in tumor tissue of nude mice knocked down by IMPDH1/2. H, GTPase activity in nude mice tumor tissue of IMPDH1/2 knockdown. I, J, Tumor morphology and weight of nude mice tumor tissue of UCA1 knockdown. K, Immunohistochemical analysis of TWIST1, IMPDH1, and IMPDH2 expression in nude mice tumor tissue of UCA1 knockdown. L, The metabolites IMP, GMP, and GTP levels in nude mice tumor tissue of UCA1 knockdown. M, UCA1, TWIST1, IMPDH1, and IMPDH2 expression levels in nude mice tissue were detected by qRT-PCR. All immunohistochemical results consist of a 10× region and a locally magnified region that is 4 times larger, scale bar 10× (left) = 200μm, scale bar 40× (right) = 50μm. Data are expressed as mean ± SEM for qRT-PCR and mean ± SD for other analyses. ns, no significance; * p<0.05; **p<0.01; ***p<0.001; **** p<0.0001.

In order to verify the results at the cellular level, we further conducted at the human bladder cancer tissue and animal levels. In human bladder urothelial carcinoma (BLCA) tissues, the proteins expression of IMPDH1, IMPDH2, and TWIST1 was up-regulated. The proportion of immunohistochemical scores of "+1" and "+2" was increased (Figure 8A), and the expression of UCA1, TWIST1, and IMPDH1 was also up-regulated at the RNA level. In contrast, the expression of IMPDH2 was not significantly elevated (Figure 8B).

Nude mice were injected subcutaneously with 5637 cells knocked down IMPDH1 and IMPDH2 to construct the subcutaneous tumor model. Tumors in the IMPDH1/2 knock-down group grew slowly and lost weight compared to the control group (Figure 8C, D). Immunohistochemistry revealed that ki67 protein expression was decreased (Figure 8E). This indicates that knockdown IMPDH1 and IMPDH2 genes can inhibit the growth rate of axillary tumors in nude mice. Interestingly, we detected higher levels of metabolites after knocking down IMPDH1 and IMPDH2 than the control group (Figure 8F). The expression of ribosome-associated pre-rRNA in tumors decreased (Figure 8G), and GTPase activity decreased (Figure 8H). When 5637 cells knocked down UCA1 were injected into nude mice for culture, the growth rate of the transplanted tumor slowed down, and the weight decreased (Figure 8I, J). The genes expression of UCA1, TWIST1, and IMPDH1 decreased at the RNA level in pRNAT-U group (Figure 8M). In UCA1 knocked down group, ki67, TWIST1, IMPDH1, and IMPDH2 proteins expression declined according to immunohistochemical (Figure 8K), and the level of metabolite GMP and GTP decreased (Figure 8L). In conclusion, we have verified most of the results at the cellular level in vivo and in animals.

## Discussion

Based on our findings we confirmed that IMPDH1 and IMPDH2 are target genes of UCA1 controlling purine metabolism in bladder cancer. The two IMPDH isoforms in vivo share 84% of the same sequence. IMPDH1 expresses in most tissues and has a housekeeping function 32-34. GTP is activated by IMPDH2 in order to promote rRNA and tRNA synthesis and enhance glioblast nuclear hypertrophy 35. The overexpression of IMPDH2 in bladder cancer tissues is highly correlated with patient's poor prognosis 36. Although the current studies concentrate on human retinal degenerative illnesses, we have discovered that IMPDH1 was also related to the prognosis of bladder cancer, and UCA1 regulated the expression of IMPDH1 and IMPDH2, which was not entirely independent on the RNA level, which have been observed in cells, clinical tissue samples, and animals but has not been observed at the protein level. In other words, when IMPDH1 expressed differently in the two groups, IMPDH2 might have a small difference when there was a large difference between the two. This suggests that the IMPDH1 and IMPDH2 genes play more complicated roles in transcription, which may result from their interactions or the control of other genes.

Pre-rRNAs and GTPases participate in several cellular processes. We unexpectedly found that when IMPDH1/2 was knocked down at the animal level, the expression of IMP, GMP, and GTP was abnormally high, which may be related to the reduced consumption of IMP, GMP, and GTP in the tumor. Since the de novo guanine nucleotide synthesis process produces more IMP, GMP, and GTP, phosphodiester bond polymerization is used to create GTP, one of the substrates for RNA polymerase, which can act as a substrate for the catalytic activity of GTPases. As a result, UCA1 accelerates the growth of tumors by controlling the metabolism of guanine nucleotides, which affects GTPase activity and the expression of pre-ribosomal RNA dependent on RNA polymerase. Researchers previously studied the effect of RNA polymerase-dependent transcription on tumor progression 37, but we have also found that elevated GTPase activity play a significant role.

For 20 years, IMPDH inhibitors have been under development 38. However, the clinical success of drug development depends on its efficacy and safety. IMPDH inhibitors have been tempered by their adverse effects at high treatment doses and variable response in cancer treatment 39. MPA and MMF inhibited the activity of IMPDH in bladder cancer cells; however, our study found that with the use of MPA and MMF, the expression of IMPDH1 and IMPDH2 proteins in bladder cancer cells increased (Supplementary Figure 2A), partially resisting the adverse effects of the decreased activity of IMPDH on tumor cells. Increased IMPDH1 and IMPDH2 expression in bladder cancer cells is likely to be driven by the strong dependence of tumor cell growth on IMPDH providing purine nucleotides and the inhibition of p53-dependent growth by the increase of IMPDH expression. Therefore, efforts to develop inhibitors targeting the expression of IMPDH and their combined use with existing inhibitors may address the adverse reactions in clinical tumor treatment caused by current IMPDH inhibitors.

Transcription factors tightly regulate nucleotide metabolic pathways. The most researched is the transcription factor c-Myc, which binds to numerous significant genes in the nucleotide metabolic pathway and directly controls key metabolic enzymes, including CAD and IMPDH 25, 31. We have used bioinformatics to predict 10 transcription factors, including c-Myc, that function as UCA1 to regulate IMPDH1 and IMPDH2, since the involvement of other transcription factors in nucleotide metabolism has received less attention. It has been discovered that UCA1 can encourage TWIST1 to bind to the IMPDH1 and IMPDH2 promoter regions, hence promoting the transcription of IMPDH1 and IMPDH2. TWIST1 has multiple binding sites with IMPDH1 and IMPDH2, and can stably regulate IMPDH1 and IMPDH2. Our study not only elucidates the molecular mechanism by which UCA1 regulates IMPDH1/2, but also confirms the role of TWIST1 in remodeling nucleotide metabolism.

**Figure 9:**
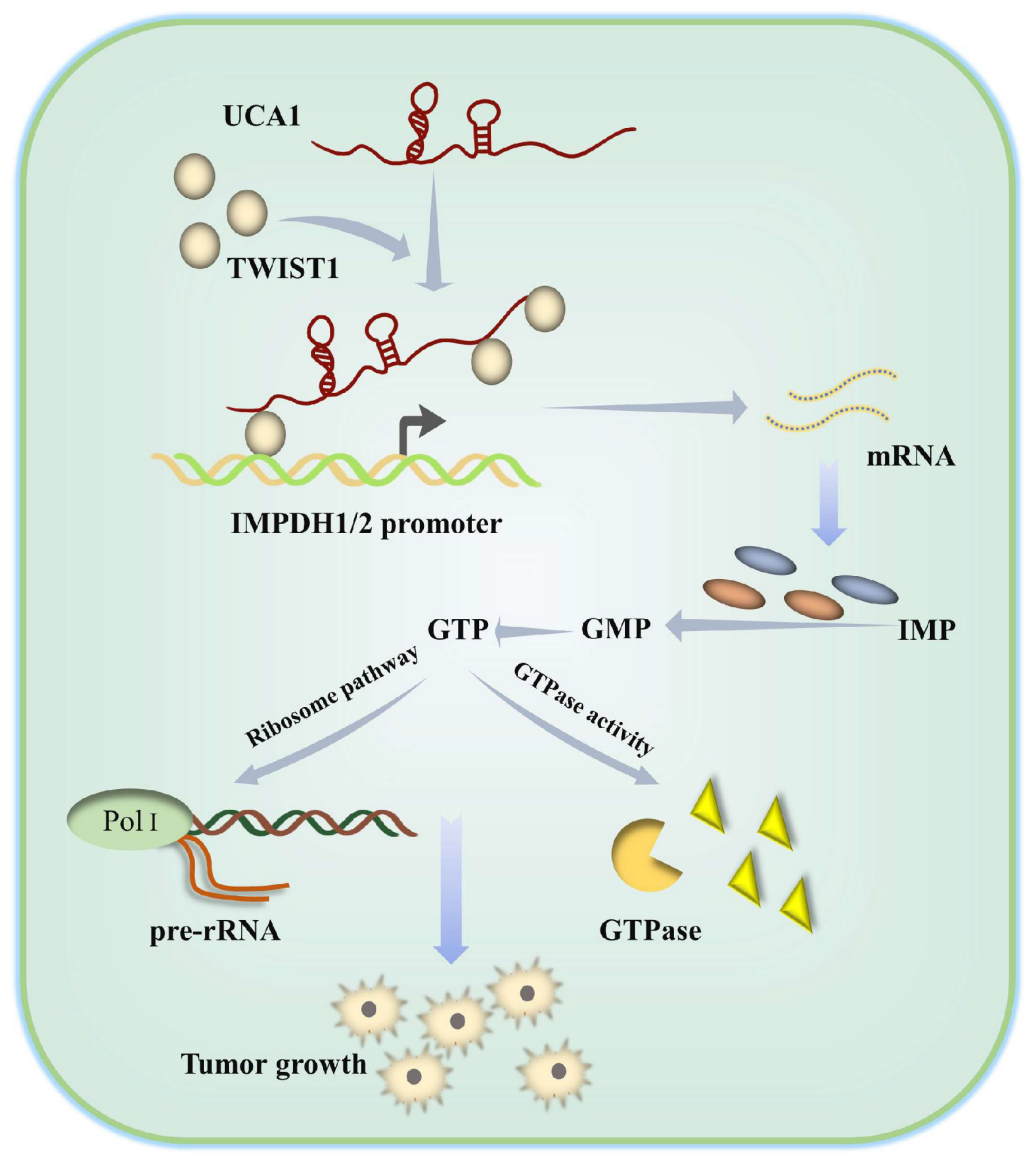
Schematic diagram showing how UCA1 participates in de novo synthesis of guanine nucleotides in bladder cancer via UCA1/TWIST1/IMPDH axes.

In summary, we have clarified the molecular mechanism by which UCA1 controls IMPDH1/2 expression via TWIST1 to change metabolite levels and promotes guanine nucleotide de novo anabolic reprogramming and bladder cancer progression (Figure 9). In addition, we have identified one of the possible reasons for the failure of the current IMPDH1/2 inhibitors, providing a reference for IMPDH1/2 inhibitor development. The development of IMPDH1/2 inhibitors and new theories about the regulatory roles of lncRNAs and transcription factors in the metabolic remodeling of bladder cancer cells contribute to the limitations of prospective treatment targets.

## Limitations

UCA1 is specifically overexpressed in bladder cancer and regulates guanine nucleotide metabolism in these tumors. However, whether it has similar effects in other non-urinary system tumors requires further experimental validation.

## Supplementary Material

Supplementary figures and tables.
