# Supplementary material for: LncRNA UCA1 Participates in De Novo Synthesis of Guanine Nucleotides in Bladder Cancer by Recruiting TWIST1 to Increase IMPDH1/2
Source: Int J Biol Sci. 2023 May 8;19(8):2599–612. doi: 10.7150/ijbs.82875 (PMC10197894; doi:10.7150/ijbs.82875)
Supplement: Supplementary file 1 — Supplementary figures and tables. [file ijbsv19p2599s1.pdf]

Supplementary Figure Legend:

Supplementary Figure 1. GSEA analysis of differential enrichment metabolic pathways in bladder cancer and adjacent tissues. (A) Pyrimidine metabolism. (B) Purine metabolism. (C) Metabolism of carbohydrates. (D) Metabolism of cofactors. (E) Metabolism of folate and pterines. (F) Tryptophan metabolism. FDR: false discovery rate.

Supplementary Figure 2. Inhibitors dose select and reverse validation of pathway molecules. (A) Select the required dose of MPA and MMF for 50% proliferation inhibition in 5637 and UMUC2 cells through western blotting. (B) UCA1 expression in 5637 and UMUC2 cells overexpressing TWIST1 was tected by qRT-PCR. (C) RNA levels of UCA1 and TWIST1 in 5637 and UMUC2 cells overexpressing IMPDH1/2 were tected by qRT-PCR. (D) Western blotting was used to test TWIST1 protein expression in 5637 and UMUC2 cells overexpressing IMPDH1/2. (E) RNA levels of UCA1 and TWIST1 in 5637 and UMUC2 cells that were knocked down with IMPDH1/2 were tested by qRT-PCR. Data are expressed as mean ± SEM for qRT-PCR and mean ± SD for other analyses. ns, no significance; * $p<0.05$; ** $p<0.01$; *** $p<0.001$; **** $p<0.0001$.

Supplementary Figure 3. Identify the efficiency of UCA1 and IMPDH1/2 in cells. (A) The efficiency of UCA1 in knockdown and overexpression cells was tested by qRT-PCR. (B-C) Western blotting results of IMPDH1 and IMPDH2 proteins expression in 5637 and UMUC2 cells overexpressing IMPDH. (D-E) Western blotting results of the expression of IMPDH1 and IMPDH2 proteins in 5637 and UMUC2 cells knockdown with IMPDH. Data are expressed as mean ± SEM for qRT-PCR and mean ± SD for other analyses. ns, no significance; * $p<0.05$; ** $p<0.01$; *** $p<0.001$; **** $p<0.0001$.

Supplementary Table Legend:

Supplementary Table 1. Primers or other sequence information.

Supplementary Table 2. Characteristics of bladder cancer patients.

Supplementary Table 3. Prediction results of transcription factors.

Supplementary Figure:

Supplementary Figure 1.

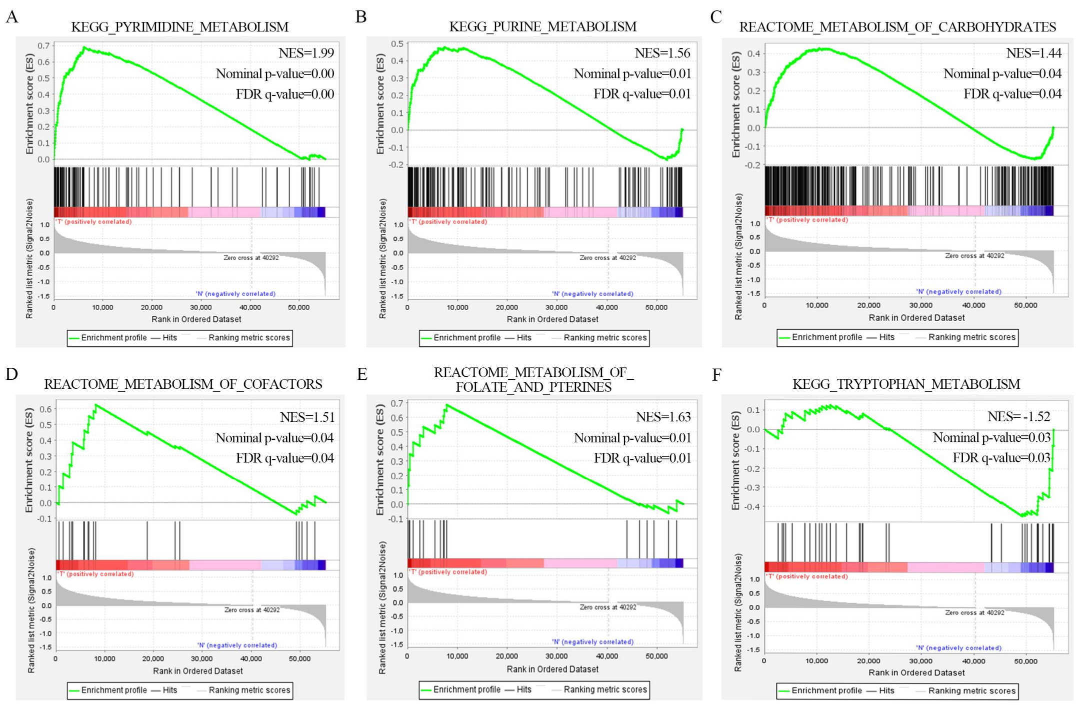

Supplementary Figure 2.

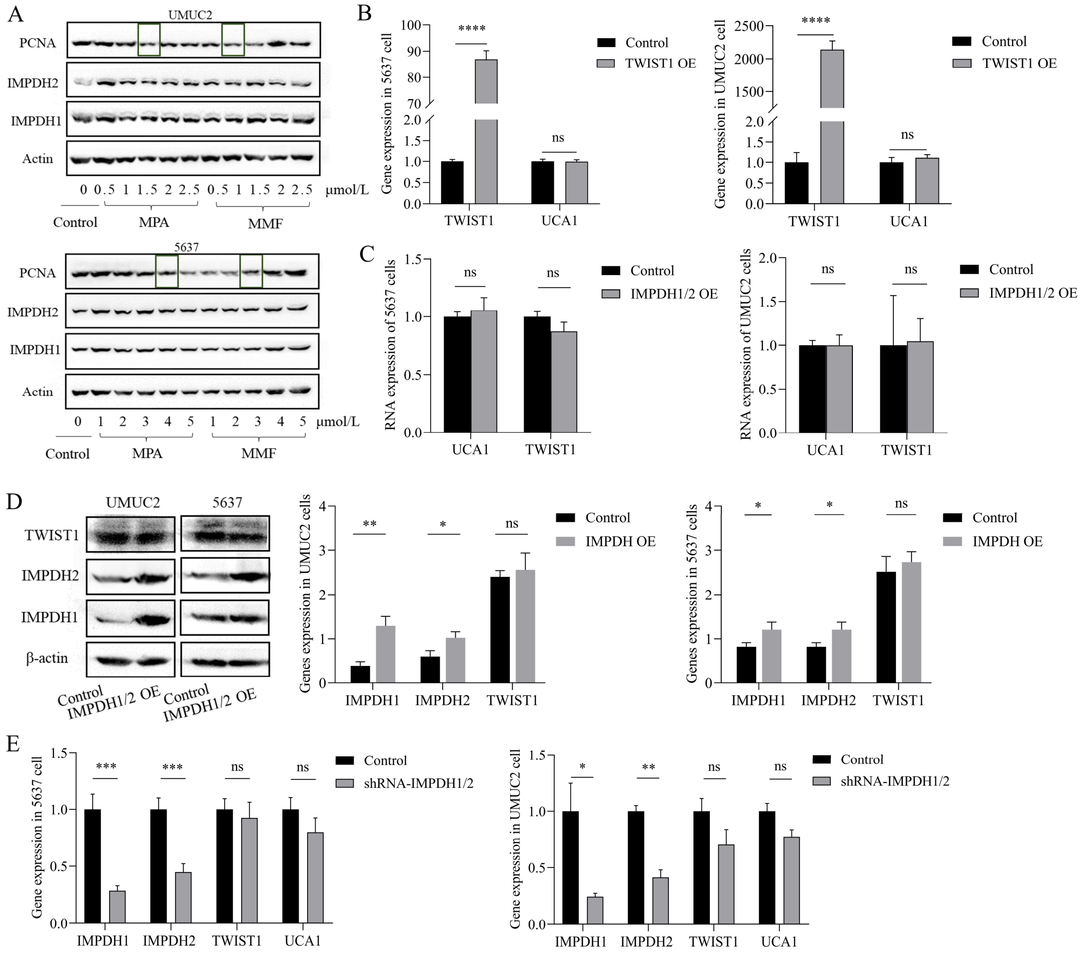

Supplementary Figure 3.

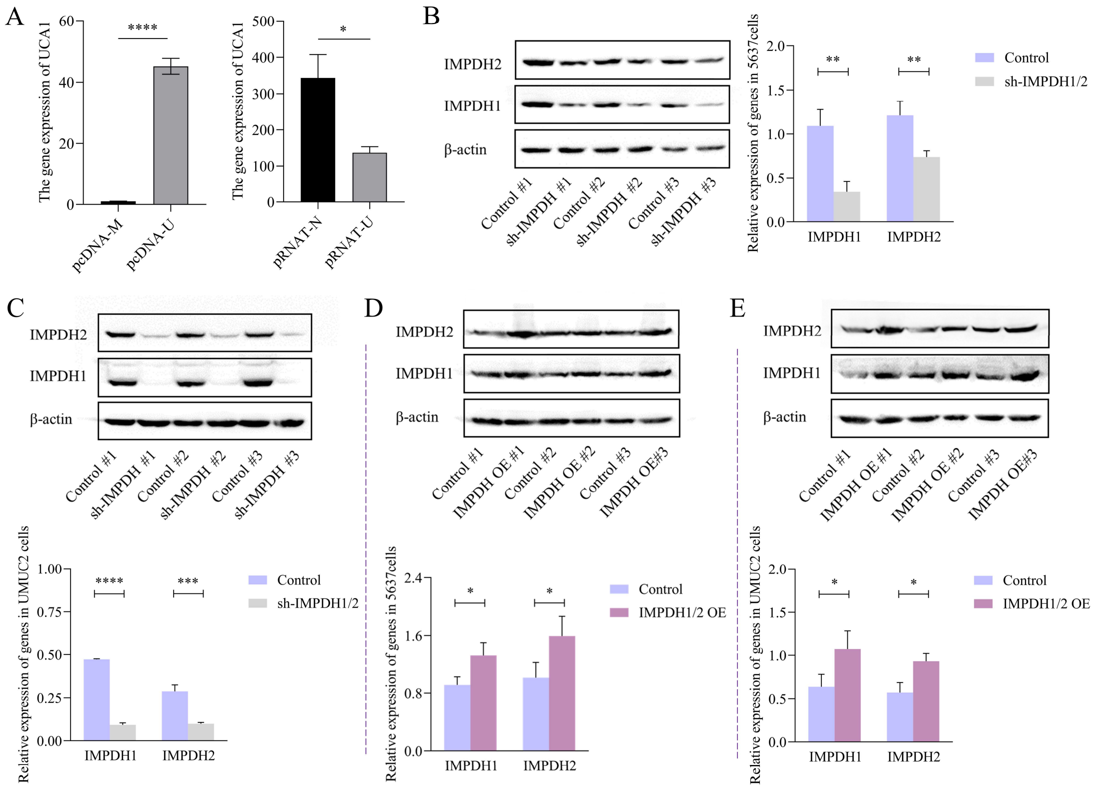

Supplementary Table:

Supplementary Table 1. Primers or other sequence information.

sh-IMPDH1/2 sequence

TGAAGAAGAACCGAGACTA

pRNAT-U6.1/Neo-Nc

5′-gatccTTCTCCGAACGTGTCACGTttcaagagaACGTGACACACGTTCGGAGAAtttt
ttggaaa-3′

pRNAT-U6.1/Neo- shUCA1.1

5′-gatccGTTAATCCAGGAGACAAAGAttcaagagaTCTTTGTCTCCTGGATTAAtttttt
gga-3′

qRCR primers (5′->3′)

β-actin:
Forward TCCCTGGAGAAGAGCTACGA
Reverse AGCACTGTGTTGGCGTACAG

GAPDH:
Forward GTCGGAGTCAACGGATTTG
Reverse TGGGTGGAATCATATTGGAA

IMPDH2:
Forward TTCTGCGGTATCCCAATC
Reverse CGAGCAAGTCCAGCCTAT

IMPDH1:
Forward CAGGGTCCGTGATGAAGC
Reverse CTCTGATGGGAGGTATTGGTT

TWIST1:
Forward TCATGGCCAACGTGCGG
Reverse CTCCATCCTCCAGACCGAGA

PRRX1:
Forward GGCTTTGGAGCGTGTCTTTG
Reverse GAGCAGGACGAGGTACGATG

ADA:
Forward CCTTCGACAAGCCCAAAGTG
Reverse GATCCCTCTCCTCCTGCCATA

ADSL:
Forward GCACAAGATTTGCACCGACA
Reverse GCATCGCACTTGAGCCAATC

ADSS:
Forward GCATTCATTGGAAATGGTGTGG
Reverse TTGTTCCTGGATACCATCAGC

ATIC:
Forward GTTGCCTGCAATCTCTATCCCT
Reverse CACTCGAGCGTGGTTTTTGG

GMPS:
Forward TCAGAAAGTTGAGAGAGGAGGGA
Reverse GGACCTGGAAATGGATGCCT

HPRT1:
Forward CCTGGCGTCGTGATTAGTGAT
Reverse TCGAGCAAGACGTTCAGTCC

PAICS:
Forward GCTGAGTATGAAGGGGATGGC
Reverse GGTTGAACAGCCAAGACCAC

PFAS:
Forward ACTCACAACTTTCCCACAGGA
Reverse TCCCAGGGCAGATTGTAACC

PPAT:
Forward TGGTGCAAGATATTACCGTGAAG
Reverse ACTGGGTTTCCTTCAGACCT

Pre-rRNA:
Forward CCGTCCGTCCGTCGTCCTCCTCGC
Reverse TGTACCGGCCGTGCGTACTTAGAC

5SrRNA:
Forward GGCGTAGAGGAACCACACCAATC
Reverse GCAGGACCTCCCCTACAGTATCG

SDHB:
Forward CTCAGGAAGGCAAGCAGCAGTATC
Reverse ATTTGTCTCCGTTCCACCAGTAGC

E2F4:
Forward GAGCAAGAACTAGACCAGCACAAGG
Reverse CCAAGAGGGTATCTCCAGCAAAGC

ATF4:
Forward TCCTGTCCTCCACTCCAGATCATTC
Reverse TCATGGCAACGTAAGCAGTGTAGTC

CHIP primer (5′->3′)

IMPDH1-1:
Forward GTCTCAGTCTCTTGACCTCGTGATC
Reverse TGGCCAACATGGCGAAACT

IMPDH1 -2:
Forward TGTTCCCTCAGACTTGTCAGACAAG
Reverse GGATGGTCTCGATCTTCTGACCTT

IMPDH1-3:
Forward ACAGAGCGAGACTCCGTCTC
Reverse GATGGTCCTCGACTTACAATGGTTGA

IMPDH1-4:
Forward GGGCTACCTCAGGGTAAATCCATC
Reverse TCCTTGCCCTCAGGCAATTTATTTCAG

IMPDH1-5:
Forward ATGTACCCTCGCTGAATCTGGG
Reverse AATCCGGGAGTCGACTGGG

IMPDH2 -1:
Forward TATGCCTCTTCTCAACTTAAACACAGC
Reverse GGAGGGGACACAGGTAGCAAT

IMPDH2 -2:
Forward TAGCTGGGCTTGGTGGCTC
Reverse GGCTGGAGTGCAGTGAGG

IMPDH2 -3:
Forward GACTCACATGAGTCCATGAGAGCC
Reverse CCACACACTACATCCTGGTCCT

IMPDH2 -4:
Forward AGTAGCTGTGCCACAATAACTTGG
Reverse TTCCGGCCTTTCACACTCTGG

IMPDH2 -5:
Forward ACACCTGAGCCAGTGGTG
Reverse GCGATGGCCTCCTGAATTTCT

IMPDH2 -6:
Forward GCGGCAGACTCCTCAGC
Reverse CTCTTGAAGATGGAAAGGGGCCA

RNA pull down primer (5′->3′)

T7 UCA1 sense sequences
Forward CCAGTACCTAATACGACTCACTATAGGGTTGACATTCTTCTGGACAATGA
Reverse ttttttttttttttttttttttttttttATCAGGCATATTAGCTTTAATGT

T7 UCA1 antisense sequences
Forward CCAATCGTAATACGACTCACTATAGGGttttttttttttttttttttttttttttttATCA
Reverse TTGACATTCTTCTGGACAATGAG

shRNA-UCA1
CCGGGTTAATCCAGGAGACAAAGACTCGAGTCTTTGTCTCCTGGATTAACTTTTTG

Supplementary Table 2. Characteristics of bladder cancer patients.

Characteristic	Patient frequency
**Total**	48
**Issue type**	
Suprapubic cystostomy	6
Bladder cancer	35
Paracancerous	17
**Gender**	
Male	36 (85.7%)
Female	6 (14.3%)
**Age**	63 (48–77)
<60	14 (33.3%)
≥60	28 (66.6%)
**TNM stage**	
I	12 (28.6%)
II	15 (35.7%)
III	13 (31%)
NA	2 (4.8%)
**Tumor stage**	
Ta	2 (4.8%)
T1	16 (38.1%)
T2	10 (23.8%)
T3-T4	11 (26.2%)
NA	3 (7.1%)
**Histopathological subtype**	
Urothelial carcinoma	48 (100%)

Supplementary Table 3. Prediction results of transcription factors.

Gene name	IMPDH1					IMPDH2					UCA1
	Binding site	Binding sequences	Binding chain	Combined with score	Correlation coefficient	Binding site	Binding sequences	Binding chain	Combined with score	Correlation coefficient	Correlation coefficient
MYC	960-971	GGCCTCGTGCCG	-	0.89	0.34	719-729	AAGCACGTCTT	+	0.82	0.16	0.33
FLI1	214-223	GCCGGAGATA	+	0.85	0.19	23-32	CCAGGAAAAA	+	0.85	0.17	0.23
MSC	78-87	AGCCCCTGTT	+	0.81	0.29	720-729	AAGACGTGCT	-	0.82	0.16	0.25
PRRX1	158-165	CAAATAAC	+	0.83	0.24	94-101	GTAGTTAC	+	0.80	0.21	0.28
TEAD2	305-317	ACCCATTCCCTTG	-	0.82	0.23	277-289	TCACAGTCCAAGC	-	0.85	0.26	0.32
IKZF1	272-283	CGGAAAGGAAGG	-	0.83	0.21	9-20	ACAAGAGAAACC	+	0.80	0.23	0.24
MSANTD3	400-409	GGCCACCCAA	+	0.82	0.46	45-54	TCACACTAAA	+	0.84	0.20	0.35
GLI2	36-50	GGGCCACCAACTTCT	+	0.87	0.17	830-844	ACCCCACACTCCCCG	-	0.83	0.15	0.25
NFIL3	361-373	ATTTATGAAAAAT	+	0.86	0.21	680-692	TCTTATGTATTCT	+	0.86	0.17	0.27
TWIST1	9-21	GATCCAGATTTCT	-	0.88	0.18	217-229	GTGTCATATGTCC	+	0.80	0.15	0.26

**Note:* the *p*- value of correlation coefficient is less than 0.0001
